# Draft Genome Sequence of *Cesiribacter andamanensis* Strain AMV16^T^, Isolated from a Soil Sample from a Mud Volcano in the Andaman Islands, India

**DOI:** 10.1128/genomeA.00240-13

**Published:** 2013-05-16

**Authors:** Sisinthy Shivaji, Sreenivas Ara, Zareena Begum, T. N. R. Srinivas, Aditya Singh, Anil Kumar Pinnaka

**Affiliations:** CSIR—Centre for Cellular and Molecular Biology, Habsiguda, Hyderabad, Andhra Pradesh, Indiaa;; CSIR—Institute of Microbial Technology, Chandigarh, Indiab

## Abstract

Here we report the 4.75-Mb genome of *Cesiribacter andamanensis* strain AMV16^T^, isolated from a soil sample from a mud volcano in the Andaman Islands, India.

## GENOME ANNOUNCEMENT

*Cesiribacter andamanensis* strain AMV16^T^ is a Gram-negative, rod-shaped, nonmotile bacterium. It was isolated from a mud sample collected from a mud volcano in the Andaman Islands, India (1). According to the 16S rRNA similarity, the members of the genus *Marivirga* are the closest phylogenetic neighbors of *Cesiribacter andamanensis* AMV16^T^, with similarities ranging from 89.9 to 90.0%.

The Roche 454 (FLX titanium) pyrosequencing platform was used to sequence the genome of *Cesiribacter andamanensis* strain AMV16^T^. The sequencing resulted in a total of 61,024,032 bases in 185,744 reads, which is 12.8× coverage of the genome. GS *De Novo* Assembler (version 2.8; F. Hoffmann-La Roche Ltd., Switzerland) was used to assemble the raw data. Assembly yielded 4,759,956 bp in 237 contigs, with all contigs ≥1,074 bp in length and the largest contig 118,810 bp in length. All sequences were above the quality score of 40, with a mean quality score of 63.12. The calculated G+C content of the genome was 54.60 mol%, which is near the experimentally determined 50.9 mol% for the bacterium (1). The annotation of the genome was performed using the Prokaryotic Genome Annotation System (Prokka) pipeline (2), tRNA was predicted by tRNAscan-SE 1.23 (3), and rRNA genes were predicted by RNAmmer 1.2 (4).

There are 4,267 predicted coding regions in the annotation of *Cesiribacter andamanensis* strain AMV16^T^, including 4 rRNA genes and 36 tRNA genes. There were 34 genes for resistance against antibiotics and toxic compounds, including 3 arsenic resistance genes, 7 genes for copper homeostasis, and 10 genes encoding cobalt-zinc-cadmium resistance. Also present in the genome were 64 genes for membrane transport, including genes for 4 ABC transporters. There were 56 genes for stress response, including 12 heat shock, 10 detoxification, and 31 oxidative stress genes. The annotation of the genome also revealed the presence of 12 genes for the metabolism of aromatic compounds.

The SEED Framework for Comparative Genomics (5) was used for functional comparison of the genome, which identified *Algoriphagus* sp. strain PR1, *Cytophaga hutchinsonii* ATCC 33406, *Spirosoma linguale* DSM 74, and *Chitinophaga pinensis* DSM 2588 as the closest neighbors of *Cesiribacter andamanensis* strain AMV16^T^, with similarity score values of 530, 429, 356, and 312, respectively. The sequencing of this bacterium from a mud volcano will assist in the elucidation of the survival methods of the bacterium under extreme conditions like high temperature.

### Nucleotide sequence accession numbers.

The draft genome sequence of *Cesiribacter andamanensis* strain AMV16^T^ has been deposited in DDBJ/EMBL/GenBank under the accession number AODQ00000000. The version described in this paper is the first version, AODQ01000000.
